# SENSE EPI reconstruction with 2D phase error correction and channel‐wise noise removal

**DOI:** 10.1002/mrm.29349

**Published:** 2022-07-25

**Authors:** Elizabeth Powell, Torben Schneider, Marco Battiston, Francesco Grussu, Ahmed Toosy, Jonathan D. Clayden, Claudia A. M. Gandini Wheeler‐Kingshott

**Affiliations:** ^1^ Queen Square MS Centre, UCL Institute of Neurology University College London London UK; ^2^ Centre for Medical Image Computing, Department of Medical Physics and Biomedical Engineering University College London London UK; ^3^ Philips Healthcare UK Guildford UK; ^4^ Radiomics Group Vall d'Hebron Institute of Oncology, Vall d'Hebron Barcelona Hospital Campus Barcelona Spain; ^5^ Developmental Imaging and Biophysics Section, Great Ormond Street Institute of Child Health University College London London UK; ^6^ Department of Brain and Behavioural Sciences University of Pavia Pavia Italy; ^7^ Brain MRI 3T Center IRCCS Mondino Foundation Pavia Italy

**Keywords:** denoising, diffusion, EPI, Nyquist ghost, SENSE

## Abstract

**Purpose:**

To develop a robust reconstruction pipeline for EPI data that enables 2D Nyquist phase error correction using sensitivity encoding without incurring major noise artifacts in low SNR data.

**Methods:**

SENSE with 2D phase error correction (PEC‐SENSE) was combined with channel‐wise noise removal using Marcenko–Pastur principal component analysis (MPPCA) to simultaneously eliminate Nyquist ghost artifacts in EPI data and mitigate the noise amplification associated with phase correction using parallel imaging. The proposed pipeline (coined SPECTRE) was validated in phantom DW‐EPI data using the accuracy and precision of diffusion metrics; ground truth values were obtained from data acquired with a spin echo readout. Results from the SPECTRE pipeline were compared against PEC‐SENSE reconstructions with three alternate denoising strategies: (i) no denoising; (ii) denoising of magnitude data after image formation; (iii) denoising of complex data after image formation. SPECTRE was then tested using high b‐value (i.e., low SNR) diffusion data (up to b=3000 s/mm${}^{2}$) in four healthy subjects.

**Results:**

Noise amplification associated with phase error correction incurred a 23% bias in phantom mean diffusivity (MD) measurements. Phantom MD estimates using the SPECTRE pipeline were within 8% of the ground truth value. In healthy volunteers, the SPECTRE pipeline visibly corrected Nyquist ghost artifacts and reduced associated noise amplification in high b‐value data.

**Conclusion:**

The proposed reconstruction pipeline is effective in correcting low SNR data, and improves the accuracy and precision of derived diffusion metrics.

## Abstract 2

Click here for author‐reader discussions

## 1 INTRODUCTION

Quantification of tissue structure or microstructure relies on an unbiased representation of the MR signal. Diffusion MRI (dMRI) provides unrivalled access to microstructural tissue properties by sensitizing the MRI signal to the self‐diffusion of water, which is influenced by the biological structures within which diffusion occurs. However, dMRI requires fast imaging and strong diffusion sensitization, resulting in two key limitations: (i) Nyquist ghost artifacts associated with the fast EPI readout, and; (ii) low signal‐to‐noise (SNR) levels at high b‐values. Moreover, residual ghosting arising from higher‐order phase errors often necessitates a more complex 2D phase correction than the routine linear phase correction implemented in scanner reconstructions.

Correcting 2D Nyquist phase errors by exploiting parallel imaging during reconstruction^1^, ^2^, ^3^, ^4^, ^5^, ^6^, ^7^ is an appealing approach in that no additional reference data are required: each dynamic image is self‐corrected using an estimate of the phase error obtained by reconstructing the odd and even k‐space lines separately. However, as splitting k‐space is effectively equivalent to doubling the acceleration factor, concomitant geometry factor dependent noise (g‐noise) amplification can reduce image quality. The PEC‐SENSE method (SENSE with phase error correction)^7^ proposed generating a 2D phase difference map from the odd and even echo reconstructions that is smoothed and then used to modify the coil sensitivity maps in the final joint SENSE reconstruction. This approach improved the conditioning of the reconstruction and lead to better SNR preservation, particularly when compared with more naïve methods such as phased array ghost elimination (PAGE).^1^ However, the method was initially validated in EPI data without diffusion weighting; a more recent study demonstrated that residual noise amplification, while negligible in nondiffusion‐weighted data, introduced visible and severe noise degradation in low SNR DWI.^8^

Denoising techniques would be a natural choice for mitigating this noise bias, and precombination denoising in particular would benefit PEC‐SENSE and other parallel‐imaging‐based 2D phase correction methods by minimizing additional noise propagation from the increased g‐factor during reconstruction. Typically, denoising is performed on the magnitude reconstructed images, either in the image or spectral domains. Spectral filtering methods decompose the data into low‐frequency (image) and high‐frequency (noise) components—often using wavelets^9^, ^10^, ^11^—but can remove small anatomical details if sharp edges contribute to the filtered high‐frequency band. Image‐domain techniques have been proposed using Gaussian filters,^12^ nonlocal means filters,^13^, ^14^, ^15^ anisotropic diffusion filters^16^, ^17^ or singular value decomposition,^18^ but can suffer from blurring or partial volume effects and may not be robust to spatially‐varying noise levels. Recently, methods based on principal component analysis (PCA) have gained attention,^19^, ^20^, ^21^, ^22^, ^23^, ^24^ demonstrating a convincing preservation of the DW signal compared with other approaches.^23^ Extensions to PCA‐based denoising methods have used random matrix theory—namely the Marcenko–Pastur law, which describes the eigenvalue distribution of random matrices—to robustly separate the signal‐dominated components from the noise‐dominated components (MPPCA).^24^ Crucially, these methods assume the noise model is independent and identically distributed (iid) Gaussian, which is not necessarily true for data that has undergone a parallel‐imaging‐based 2D phase correction. Current PCA‐based denoising strategies therefore face two challenges in compensating for noise amplification in PEC‐SENSE data: (i) spatial correlations may be introduced during phase correction that violate the iid Gaussian noise statistics if denoising is performed after image formation, and; (ii) increased noise levels may introduce a noise floor and minimum measurable signal that significantly biases low SNR data—even after postprocessing corrections for Rician bias^25^—if denoising is performed after signal rectification.^26^ Denoising complex channel data prior to Nyquist phase error correction and image formation could alleviate both issues and lead to superior noise statistics; indeed, promising reductions in noise floor levels have been demonstrated by denoising channel data.^27^

In this work, the efficacy of MPPCA denoising prior to image formation in mitigating g‐noise amplification associated with parallel‐imaging‐based Nyquist phase error correction is investigated. A new pipeline—SENSE with 2D PhasE CorrecTion and channel‐wise noise REmoval, coined SPECTRE^28^—is proposed for robust elimination of Nyquist ghost artifacts and alleviation of g‐noise amplification in high b‐value DWI. The accuracy and precision of diffusion metrics obtained using the new pipeline are evaluated in phantom data; the pipeline is then further explored in a small cohort of healthy subjects.

## 2 METHODS

### 2.1 Data acquisition

The FUNSTAR phantom^29^, ^30^ and four healthy volunteers (one female; mean age 35±5 years) were imaged on a 3T Philips Ingenia CX system using the vendor's 32‐channel headcoil. Written informed consent was provided by each subject before imaging in accordance with local ethics guidance. Multishell diffusion‐weighted EPI (DW‐EPI) were acquired for the subjects with TR/TE=5000/88 ms, resolution 2×2×2 mm${}^{3}$, matrix size 112×110, 20 slices, b‐values b=1000, 2000, 3000 s/mm${}^{2}$ along 30 isotropically‐distributed directions and 9 b=0 volumes. Phantom DW‐EPI were acquired with TR/TE=2000/132 ms, resolution 2×2×2 mm${}^{3}$, matrix size 120×118, 10 slices, 20 b‐values evenly spaced every 200 s/mm${}^{2}$ between 200and4000 s/mm${}^{2}$ each along 6 directions and 7 b=0 volumes. All data were acquired with SENSE factor R=2, no partial Fourier encoding and no slice gap. An additional b=0 volume with opposing phase‐encoding blips was acquired for each subject to allow for susceptibility‐induced distortion correction. Total scan time in volunteers was 13 min. Complex data from individual channels were obtained in the hybrid $\left(x,k_{y}\right)$ domain after the vendor's 1D phase correction, prior to any filtering. Full field‐of‐view (FOV) coil sensitivity profiles were also exported. All subsequent data processing was performed offline in Matlab 2019b (The MathWorks). Image space data were obtained by 1D Fourier transformation along the phase‐encoded y‐axis.

The FUNSTAR phantom was additionally imaged using a DW sequence with conventional spin echo (SE) readout; the manufacturer's reconstruction was used and no denoising was performed. Acquisition parameters were: TR/TE=4000/81 ms, resolution 3×3×3 mm${}^{3}$, matrix size 64×64, 24 slices, b‐values b=500, 1000 s/mm${}^{2}$ each along 6 directions and 1 b=0 volume. These data provided a baseline estimate of the phantom's diffusivity independent of EPI distortion and ghosting.

### 2.2 SPECTRE pipeline

The individual channel images (S) and sensitivity profiles (C) were first prewhitened to ensure that noise statistics in each coil were iid Gaussian, giving $S_{w}$ and $C_{w}$. Using a noise‐only region at the top of each coil image, a noise covariance matrix Ψ was computed. The noise decorrelation matrix was defined as $D={\left(\Psi_{L}\Psi_{L}^{H}\right)}^{-1}$, which is the inverse Cholesky decomposition of the lower triangle of Ψ ($\Psi_{L}$). Coil image and sensitivity profile data were then modified according to $S_{w}=DS$ and $C_{w}=DC$, respectively.

**FIGURE 1:**
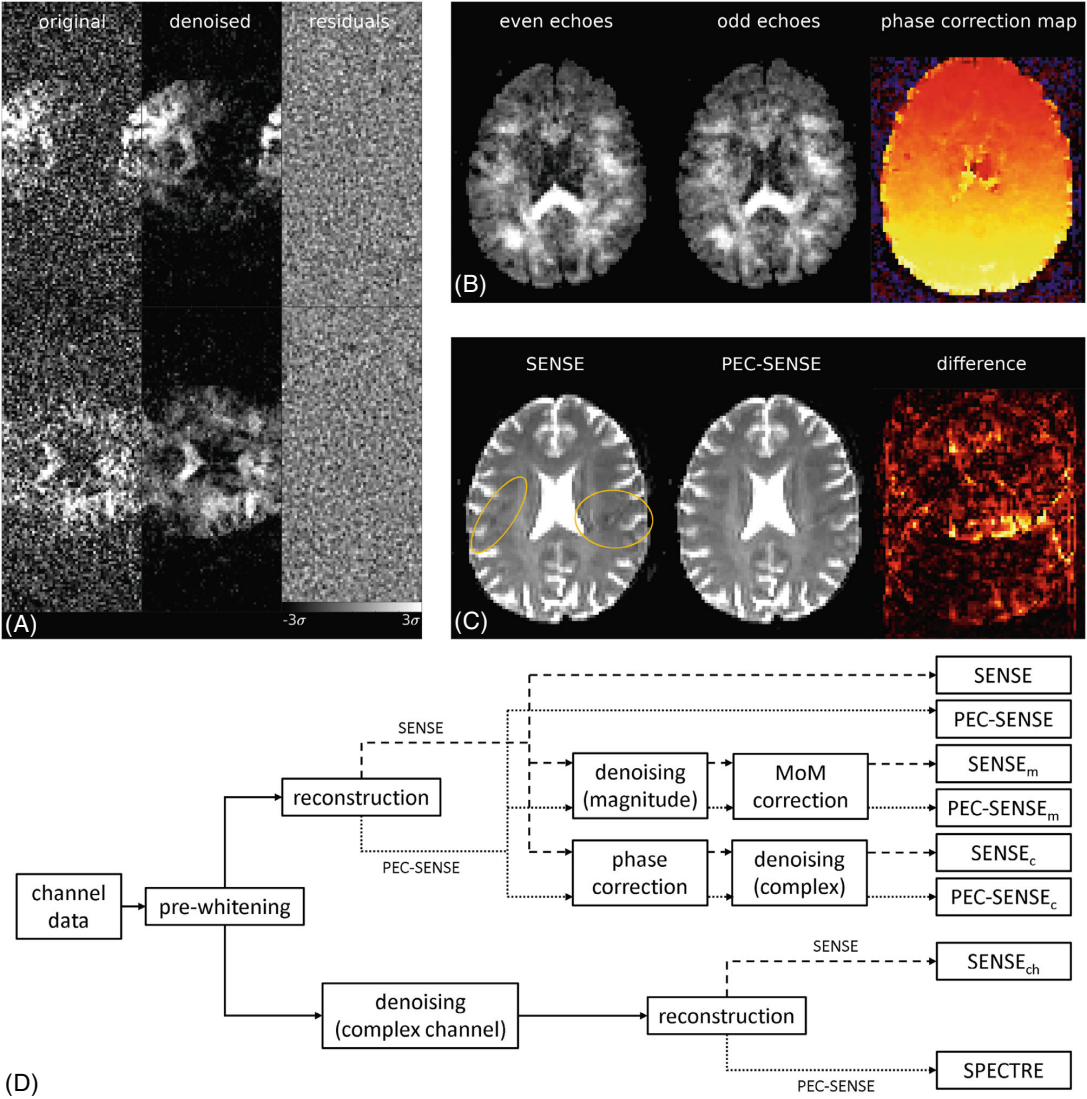
SPECTRE pipeline. (A) Representative data from two channels before (left) and after (center) denoising. Residuals between the noisy and denoised data (right) indicate that no anatomical information was lost during denoising, even in areas of aliased signal. Note the FOV is reduced owing to a parallel imaging factor of 2. (B) The even (left) and odd (center) echoes used to generate a phase correction map (right) per EPI. (C) SENSE and PEC‐SENSE reconstructions. The ghost artifact present in the SENSE reconstruction (yellow outline) was corrected in the PEC‐SENSE reconstruction. The difference between PEC‐SENSE and SENSE reconstructions highlights areas of signal aliasing that were removed by the phase correction. (D) Processing stages of each pipeline. MoM, method of moments correction.

The adjusted complex channel data $S_{w}$ were independently denoised using the MPPCA method^24^ with a sliding 5×5×5 kernel, giving $S_{w}^{\prime }$. Finally, the PEC‐SENSE reconstruction framework^7^ was used for Nyquist phase error correction and image formation. A graphical representation of the pipeline is given in Figure 1.

### 2.3 Alternative pipelines

Denoised complex channel data were also reconstructed using the standard SENSE method, denoted ${\text{SENSE}}_{\text{ch}}$ (using this nomenclature, SPECTRE corresponds to ${\text{PEC‐SENSE}}_{\text{ch}}$). Channel data were prewhitened as in the SPECTRE pipeline.

SENSE and PEC‐SENSE reconstructions were additionally generated using the original channel data (i.e., without initial channel‐wise denoising), with denoising instead performed after image formation using both magnitude and complex data; these pipelines are respectively indicated by the subscripts *m* and *c*. The method of moments correction^25^ was used to account for Rician noise bias after denoising the magnitude data. Phase artifacts in the complex data—which may contribute additional significant components in the MPPCA denoising method—were removed prior to denoising using the decorrelated phase filtering algorithm.^31^, ^32^ All denoising was performed using a 5×5×5 kernel.

The SENSE reconstructions were used to provide a baseline for SNR measurements‐the SNR in SENSE will always be equal or higher than in PEC‐SENSE as the same reconstruction problem is solved but with a lower effective g‐factor‐and to indicate the level of residual Nyquist ghosting after the vendor's 1D phase correction.

### 2.4 Accuracy and precision in phantom data

Diffusion tensor fitting was performed in the DW‐SE and DW‐EPI datasets using an iteratively re‐weighted least squares estimator^33^ in volumes with b≤1000 s/mm${}^{2}$. The SPECTRE pipeline was compared against the alternative pipelines using the accuracy and precision of mean diffusivity (MD) estimates within the phantom as benchmarks of performance; the MD estimated in the DW‐SE data was taken as the ground truth value. Accuracy was defined as the mean voxel‐wise error relative to the ground truth value, and precision as the coefficient of variation (CoV) across voxels. The SNR was calculated for each pipeline using the multiple replicas of the b=0 data.

### 2.5 Evaluation in volunteer data

For each pipeline, the reconstructed volunteer data were corrected to remove Gibbs ringing artifacts,^34^ susceptibility distortions,^35^, ^36^ and eddy current and motion artifacts^37^ using MRtrix.^33^ $B_{1}$ bias field inhomogeneities were corrected using FSL FAST.^38^ The diffusion tensor (DT) and diffusion kurtosis tensor (DKT) models were fitted to each dataset using MRtrix, with the DT fit constrained to data with b≤1000 s/mm${}^{2}$. The precision of parameter fits was evaluated using the normalized root‐mean‐squared error (NRMSE) between the data and model predictions.

## 3 RESULTS

### 3.1 Accuracy and precision in phantom data

**FIGURE 2:**
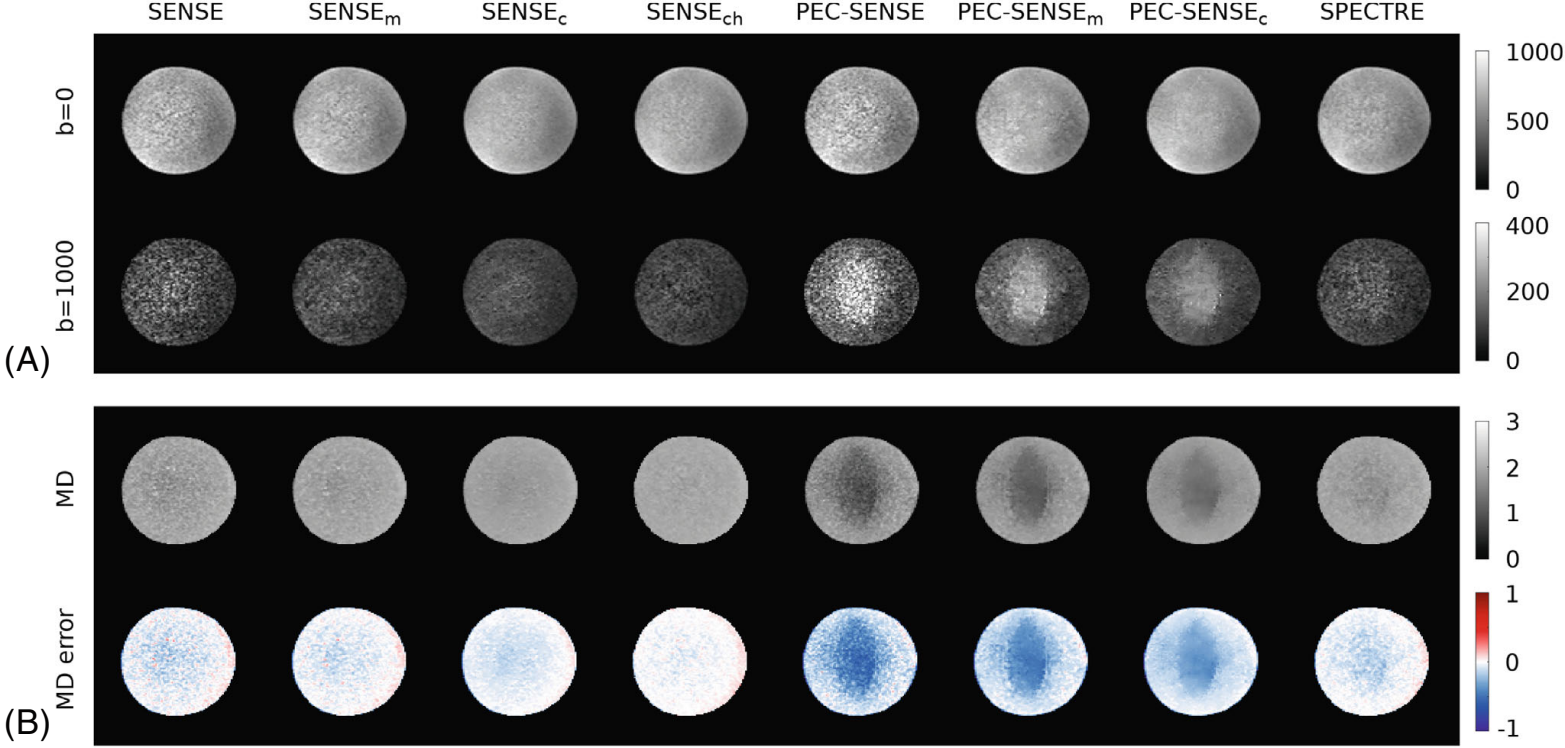
Phantom data. (A) Reconstructed phantom images at b=0 and b=1000 s/mm${}^{2}$. (B) MD maps $(\times 10^{-3}$ mm${}^{2}$/s) and the error relative to the ground truth value. Noise amplification introduced by the 2D phase error correction and corresponding biases in MD are progressively improved by the denoising strategies from left (PEC‐SENSE) to right (SPECTRE); minimal noise amplification remains after channel‐wise noise removal in the SPECTRE pipeline

Figure 2 shows example phantom data from each reconstruction pipeline. The effects of g‐noise amplification were prominent in the PEC‐SENSE, ${\text{PEC‐SENSE}}_{m}$ and ${\text{PEC‐SENSE}}_{c}$ data, most notably as regions of artificially inflated signal in the high b‐value DW data (Figure 2A). The benefits of denoising complex data prior to signal rectification were seen in the SPECTRE reconstruction, where, qualitatively, the noise floor artifacts were greatly reduced.

Table 1 presents average SNR estimates in the b=0 data for all reconstruction pipelines. SNR was lower in all PEC‐SENSE reconstructions compared with the corresponding SENSE reconstructions. SNR was most improved by the SPECTRE pipeline when phase correction was performed, showing a 127% increase over the PEC‐SENSE data.

**TABLE 1:** Quantitative values in phantom and in vivo data

	Phantom			In vivo	
	SNR	MD mean (SD) ($\times 10^{-3}$ mm${}^{2}$/s)	MD CoV (%)	SNR	NRMSE (%)
SENSE	11.5	1.85 (0.16)	8.5	25.6	8.1
${\text{SENSE}}_{m}$	13.5	1.86 (0.15)	7.8	32.4	6.0
${\text{SENSE}}_{c}$	16.9	1.84 (0.13)	7.2	36.3	5.2
${\text{SENSE}}_{\text{ch}}$	26.6	1.92 (0.07)	3.8	43.6	5.3
PEC‐SENSE	9.0	1.54 (0.31)	19.9	21.4	9.8
${\text{PEC‐SENSE}}_{m}$	12.6	1.57 (0.28)	18.1	27.8	7.1
${\text{PEC‐SENSE}}_{c}$	15.7	1.62 (0.25)	15.8	33.4	6.1
SPECTRE	20.4	1.85 (0.12)	6.4	36.4	6.3
DW‐SE (reference)	‐	2.00 (0.10)	5.0	‐	‐

*Note*: For the phantom data (left), the SNR, MD mean and standard deviation across the phantom, and MD CoV are shown, along with the ground truth MD measured in the reference DW‐SE. For the in vivo data (right), the SNR and NRMSE in white matter averaged over all subjects is shown.

Noise amplification in the PEC‐SENSE, ${\text{PEC‐SENSE}}_{m}$, and ${\text{PEC‐SENSE}}_{c}$ reconstructions severely impacted the accuracy of MD estimates (Figure 2B and Table 1): MD was underestimated by 23%, 22%, and 19% respectively, with regions coinciding with the noise floor artifacts in high b‐value data most affected. MD estimates in SPECTRE were within 8% of the ground truth value. The precision of MD estimates (Table 1) related to the complexity of the denoising strategy: the CoV was highest without denoising, improved (reduced) with magnitude and complex denoising of reconstructed data, and was lowest with denoising of complex channel data.

### 3.2 Evaluation in volunteer data

**FIGURE 3:**
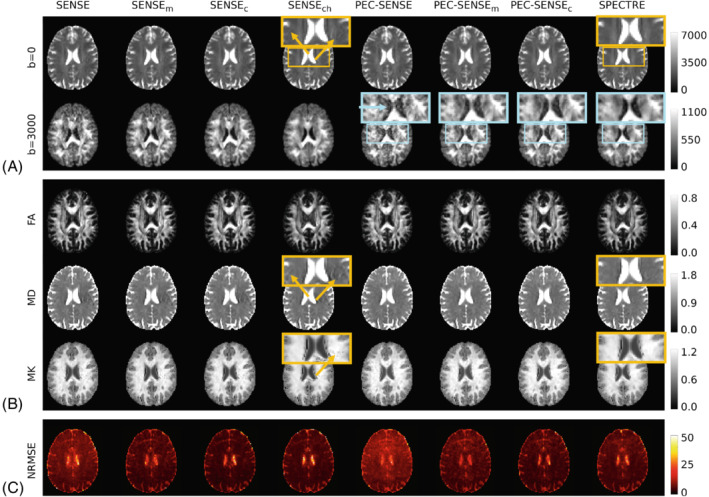
In vivo data. (A) Reconstructed data from a representative subject for each pipeline at b=0 and b=3000 s/mm${}^{2}$. Yellow boxes and arrows highlight areas of ghost artifacts in the SENSE data, which are qualitatively well corrected in the PEC‐SENSE data. Blue boxes and arrows highlight the progressive reduction in noise amplification in the PEC‐SENSE data by the denoising strategies from left (PEC‐SENSE) to right (SPECTRE). (B) Parameter maps showing FA, MD $(\times 10^{-3}$ mm${}^{2}$/s), and MK in each pipeline. Yellow boxes again highlight the translation of ghost artifacts into parameter maps derived from SENSE reconstructions, which are less apparent in parameter maps derived from PEC‐SENSE reconstructions. (C) NRMSE (%) between the data and DKI model fit

Figure 3 shows image reconstructions and parameter maps for a representative subject. Nyquist ghosting was evident in the standard SENSE reconstructions (Figure 3A) and translated into notable errors in the parameter maps (Figure 3B). Ghosting was visibly reduced by the phase error correction in PEC‐SENSE‐based reconstructions, but significant g‐noise amplification was observed at higher b‐values without complex channel denoising (Figure 3A). Qualitatively, noise levels in the high b‐value SPECTRE data were reduced in line with the standard SENSE reconstruction. The SNR at b=0 is given in Table 1. The greatest SNR gain was observed in the SPECTRE reconstructions (70% increase over the standard PEC‐SENSE data), while gains of 56% and 30% were recorded in the ${\text{PEC‐SENSE}}_{c}$ and ${\text{PEC‐SENSE}}_{m}$ data respectively.

The precision of each pipeline is indicated by voxel‐wise maps of the NRMSE between the data and DKI model fit in Figure 3C; mean values in white matter over all subjects are given in Table 1. Comparable precision (NRMSE ∼ 6%) was observed in the ${\text{PEC‐SENSE}}_{c}$ and SPECTRE data.

**FIGURE 4:**
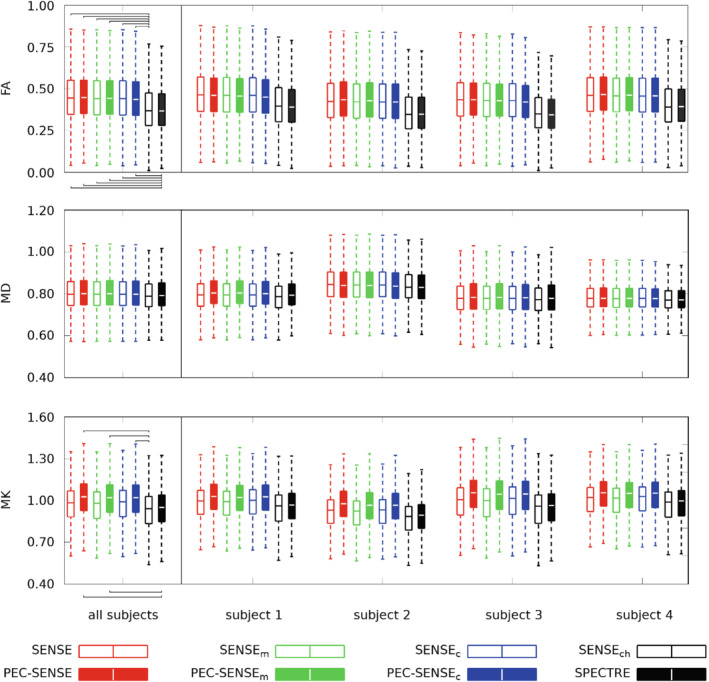
Parameter distributions in vivo for each pipeline. Values in white matter for FA, MD $(\times 10^{-3}$ mm${}^{2}$/s), and MK are shown averaged across subjects (left) and for each subject individually (right). Brackets indicate significant differences in parameter distributions between reconstructions (P<0.05)

Boxplots in Figure 4 compare the distribution of parameter estimates in each subject between the pipelines; the data are tabulated in the Supporting Information (Tables S1 – S3 ). No significant differences in fractional anisotropy (FA) were found between the phase‐corrected and standard SENSE reconstructions with equivalent denoising strategies; however, FA was significantly lower (14.5% on average) in the reconstructions denoised prior to image formation (i.e., ${\text{SENSE}}_{\text{ch}}$ and SPECTRE pipelines) compared with the data denoised after image formation or not denoised at all (i.e., all other reconstructions). Mean kurtosis (MK) tended toward higher values in the phase‐corrected data compared with the equivalently‐denoised data without phase correction; however, significant differences were only detected between the PEC‐SENSE‐based reconstructions without complex channel denoising and the ${\text{SENSE}}_{\text{ch}}$ and SPECTRE reconstructions.

## 4 DISCUSSION

Nyquist ghost correction is important in order to minimize biases in quantitative parameter maps; however, it was shown here that phase error corrections based on parallel imaging may not translate well to DW data due to noise amplification that disproportionately affects high b‐value images. This study demonstrates that, by denoising raw channel data obtained from coil elements prior to image formation, PEC‐SENSE ghost correction can be applied in high b‐value data without incurring major biases in the accuracy and precision of parameter estimates. Denoising complex channel data has previously been proposed to achieve higher resolution in DWI;^27^ here we combine the lower noise floor and SNR gains of this denoising strategy to mitigate g‐noise amplification concomitant with the phase error correction in PEC‐SENSE.

The Rician noise floor is a common limiting factor in acquiring high b‐value magnitude DWI, as the signal can often fall below the noise floor. Image reconstruction using PEC‐SENSE compounds the issue owing to g‐noise amplification associated with the phase error correction. Although techniques for Rician bias correction have been proposed,^25^ they may only offer moderate benefits. In this context, complex denoising would seem advisable. The phantom data in this study demonstrated the benefits of complex denoising, particularly when performed on raw channel data before image formation with phase error correction. Qualitatively, the Rician bias evident in the phase‐corrected high b‐value data was removed by the SPECTRE pipeline (Figure 2A), while quantitatively the accuracy and precision of MD estimates were improved (Figure 2B). SNR was also improved (Table 1). The improved performance of the SPECTRE pipeline compared with the alternative PEC‐SENSE‐based methods may be attributed to two primary factors: (i) denoising raw channel data avoided the introduction of spurious significant components into the MPPCA denoising— arising, for example, from spatial correlations propagated by the coil sensitivity maps into the phase error correction map and subsequent reconstructions—that may degrade the performance of the algorithm; and (ii) reducing noise levels prior to image formation subsequently limited noise propagation during reconstruction, therefore lowering the noise floor and minimum measurable signal.

In volunteer data, the impact of complex channel denoising was most evident in the estimation of FA, where a significant decrease compared with all other phase‐corrected pipelines was observed. There is a well‐documented dependency of FA estimation on SNR,^26^, ^39^ attributed at moderate b‐values (i.e., when noise floor effects are not dominant) to eigenvalue repulsion; as MD estimates were constant across the pipelines (Figure 4), it can be assumed that signal variance was the dominant factor as opposed to noise floor effects in this work also. It has been shown that, at b=1000 s/mm${}^{2}$, FA is overestimated at low SNR and converges to the true value as SNR increases.^26^ This effect may explain our results, where increased SNR values and lower (potentially more accurate) FA estimates were observed in the SPECTRE pipeline. The result was also replicated in the ${\text{SENSE}}_{\text{ch}}$ data. However, as the SNR gains at b=0 of SPECTRE over ${\text{PEC‐SENSE}}_{c}$ were small (under 10%; Table 1), it could be the noise statistics in the higher b‐value data that were driving this result. For example, noise removal in the higher b‐value data may be significantly more effective when using MPPCA on raw channel data than on data after image formation. As SNR estimates were not possible for the high b‐value data in this study owing to a lack of multiple replicas, this warrants future exploration.

The SPECTRE pipeline also qualitatively reduced the noise floor effects observed in estimates of MK. Highly attenuated signals at b=3000 s/mm${}^{2}$ are more susceptible to the noise floor effects, which cause an overestimation of MK values,^40^, ^41^ a trend that was observed in the higher MK estimates of PEC‐SENSE, ${\text{PEC‐SENSE}}_{m}$ ${\text{PEC‐SENSE}}_{c}$ pipelines compared to SENSE, ${\text{SENSE}}_{m}$ and ${\text{SENSE}}_{c}$ respectively. Qualitatively, MK estimates were only marginally increased in SPECTRE compared with ${\text{SENSE}}_{\text{ch}}$, suggesting that the noise floor amplification from the 2D phase error correction was largely mitigated by complex channel denoising prior to image formation.

The primary benefit of SPECTRE over ${\text{SENSE}}_{\text{ch}}$ was the correction of the Nyquist ghost artifact. Although ghost artifacts were only minimally apparent in the image data, more significant artifacts were observed in the MD and MK parameter maps in data without phase error correction (Figure 3) due to the inherent averaging effect of the model‐fitting procedure.

Improved noise reduction may be possible in the SPECTRE pipeline by removing physiological fluctuations. To avoid the risk of interfering with the sensitivity‐encoding phase, physiological phase correction was not performed on the raw channel data in this work; however, data complexity would be reduced by removing physiologically‐induced phase, and approaches for this should be explored in future work. The lack of physiological fluctuations in phantom data may explain the comparatively superior SNR performance of SPECTRE over ${\text{PEC‐SENSE}}_{c}$ in the phantom data compared with the volunteer data (Table 1).

A key limitation of this study is the lack of ground truth data in vivo. While phantom experiments confirmed that SPECTRE reduced biases in MD estimates (both higher accuracy and precision were achieved), the accuracy of the pipeline in human volunteers could not be characterized quantitatively. Future work could consider creating a silver‐standard reference using a high SNR group‐wise average data set; however, requirements for robust Nyquist phase error correction make obtaining these data challenging.

It is noted here that the use of the vendor's 1D phase correction is not a requirement in the SPECTRE pipeline, but was used in this study to highlight the necessity of higher‐order phase corrections based on the residual ghosting following 1D phase correction. Simplifying the pipeline and removing this step from the preprocessing may further improve results in future work.

The improvement in image quality obtained using the SPECTRE pipeline has been shown here to limit biases in parameter maps derived from complex, higher‐order diffusion models such as DKI, which rely on high b‐value (low SNR) data; however, the method is not limited to DWI applications, and would benefit other applications with redundant data that utilize an EPI readout, such as functional MRI, arterial spin labeling, or more advanced quantitative protocols for relaxometry and magnetization transfer imaging.

## 5 CONCLUSIONS

SPECTRE, a new method for Nyquist phase error correction that combines MPPCA denoising of complex‐valued channel data with PEC‐SENSE image formation, is shown to be feasible and robust to g‐noise amplification in high b‐value, low SNR data.

## DISCLOSURES

AstraZeneca was not involved in any aspect concerning this study: it has not influenced the analysis of the data, the interpretation of the results, and the decision to submit the manuscript for publication.

## Supporting information




**Table S1**. Comparison of FA between pipelines.
**Table S2**. Comparison of MD between pipelines.
**Table S3**. Comparison of MK between pipelines.
