# Supplementary material for: SENSE EPI reconstruction with 2D phase error correction and channel‐wise noise removal
Source: Magn Reson Med. 2022 Jul 25;88(5):2157–66. doi: 10.1002/mrm.29349 (PMC9545987; doi:10.1002/mrm.29349)
Supplement: Supplementary file 1 — Table S1. Comparison of FA between pipelines. Table S2. Comparison of MD between pipelines. Table S3. Comparison of MK between pipelines. [file MRM-88-2157-s001.pdf]

Supporting Figures and Tables

Table S1. Comparison of FA between pipelines.

The mean and SD of white matter FA over all subjects for each pipeline is shown in the leftmost column. On the right, mean white matter FA values in individual subjects are compared between pipelines using a two-tailed T-test; significant P-values ($P < 0.05$) are marked with an asterisk.

		p-values						
	mean (SD)	SENSE	PEC-SENSE	$SENSE_m$	PEC-$SENSE_m$	$SENSE_c$	PEC-$SENSE_c$	$SENSE_{ch}$
SENSE	0.455 (0.019)	-	-	-	-	-	-	-
PEC-SENSE	0.457 (0.017)	0.901	-	-	-	-	-	-
$SENSE_m$	0.452 (0.019)	0.800	0.699	-	-	-	-	-
PEC-$SENSE_m$	0.452 (0.018)	0.804	0.701	0.989	-	-	-	-
$SENSE_c$	0.452 (0.020)	0.760	0.661	0.957	0.945	-	-	-
PEC-$SENSE_c$	0.447 (0.020)	0.493	0.409	0.660	0.643	0.701	-	-
$SENSE_{ch}$	0.389 (0.026)	**0.003***	**0.002***	**0.004***	**0.004***	**0.004***	**0.007***	-
SPECTRE	0.385 (0.026)	**0.002***	**0.002***	**0.003***	**0.003***	**0.003***	**0.005***	0.842

Table S2. Comparison of MD between pipelines.

The mean and SD of white matter MD over all subjects for each pipeline is shown in the leftmost column. On the right, mean white matter MD values in individual subjects are compared between pipelines using a two-tailed T-test; there were no significant differences.

		p-values						
	mean (SD) [$\times 10^{-3}$ mm^2/s]	SENSE	PEC-SENSE	SENSE$_\mathrm{m}$	PEC-SENSE$_\mathrm{m}$	SENSE$_\mathrm{c}$	PEC-SENSE$_\mathrm{c}$	SENSE$_\mathrm{ch}$
SENSE	0.813 (0.032)	-	-	-	-	-	-	-
PEC-SENSE	0.820 (0.028)	0.731	-	-	-	-	-	-
SENSE$_\mathrm{m}$	0.813 (0.032)	0.990	0.720	-	-	-	-	-
PEC-SENSE$_\mathrm{m}$	0.820 (0.028)	0.728	0.996	0.718	-	-	-	-
SENSE$_\mathrm{c}$	0.813 (0.032)	0.983	0.714	0.993	0.711	-	-	-
PEC-SENSE$_\mathrm{c}$	0.818 (0.028)	0.800	0.921	0.789	0.918	0.783	-	-
SENSE$_\mathrm{ch}$	0.806 (0.033)	0.725	0.484	0.734	0.483	0.740	0.539	-
SPECTRE	0.814 (0.029)	0.975	0.740	0.964	0.738	0.957	0.814	0.690

Table S3. Comparison of MK between pipelines.

The mean and SD of white matter MK over all subjects for each pipeline is shown in the leftmost column. On the right, mean white matter MK values in individual subjects are compared between pipelines using a two-tailed T-test; significant P-values ($P < 0.05$) are marked with an asterisk.

		p-values						
	mean (SD)	SENSE	PEC-SENSE	SENSE_m	$\text{PEC-SENSE}_\text{m}$	SENSE_c	$\text{PEC-SENSE}_\text{c}$	SENSE_ch
SENSE	0.952 (0.039)	-	-	-	-	-	-	-
PEC-SENSE	1.004 (0.035)	0.058	-	-	-	-	-	-
SENSE_m	0.947 (0.046)	0.848	0.059	-	-	-	-	-
$\text{PEC-SENSE}_\text{m}$	0.992 (0.035)	0.126	0.582	0.119	-	-	-	-
SENSE_c	0.953 (0.040)	0.968	0.065	0.821	0.139	-	-	-
$\text{PEC-SENSE}_\text{c}$	1.035 (0.108)	0.146	0.554	0.133	0.414	0.151	-	-
SENSE_ch	0.906 (0.042)	0.109	**0.006***	0.176	**0.011***	0.107	**0.042***	-
SPECTRE	0.918 (0.041)	0.213	**0.010***	0.320	**0.019***	0.207	0.058	0.646
